# Body Temperature Frequency Distributions: A Tool for Assessing Thermal Performance in Endotherms?

**DOI:** 10.3389/fphys.2021.760797

**Published:** 2021-10-14

**Authors:** D.L. Levesque, J. Nowack, J.G. Boyles

**Affiliations:** ^1^School of Biology and Ecology, University of Maine, Orono, ME, United States; ^2^School of Biological and Environmental Sciences, Liverpool John Moores University, Liverpool, United Kingdom; ^3^Cooperative Wildlife Research Laboratory, Center for Ecology, and School of Biological Sciences, Southern Illinois University, Carbondale, IL, United States

**Keywords:** heterothermy, mammal, torpor, additive quantile regression, skew, acrophase, scotophase

## Abstract

There is increasing recognition that rather than being fully homeothermic, most endotherms display some degree of flexibility in body temperature. However, the degree to which this occurs varies widely from the relatively strict homeothermy in species, such as humans to the dramatic seasonal hibernation seen in Holarctic ground squirrels, to many points in between. To date, attempts to analyse this variability within the framework generated by the study of thermal performance curves have been lacking. We tested if frequency distribution histograms of continuous body temperature measurements could provide a useful analogue to a thermal performance curve in endotherms. We provide examples from mammals displaying a range of thermoregulatory phenotypes, break down continuous core body temperature traces into various components (active and rest phase modes, spreads and skew) and compare these components to hypothetical performance curves. We did not find analogous patterns to ectotherm thermal performance curves, in either full datasets or by breaking body temperature values into more biologically relevant components. Most species had either bimodal or right-skewed (or both) distributions for both active and rest phase body temperatures, indicating a greater capacity for mammals to tolerate body temperatures elevated above the optimal temperatures than commonly assumed. We suggest that while core body temperature distributions may prove useful in generating optimal body temperatures for thermal performance studies and in various ecological applications, they may not be a good means of assessing the shape and breath of thermal performance in endotherms. We also urge researchers to move beyond only using mean body temperatures and to embrace the full variability in both active and resting temperatures in endotherms.

## Introduction

Thermoregulation and thermal sensitivity are vital to how an individual, population or species interacts with the environment. This has never been more true than under current shifts in environmental conditions associated with climate change (Huey et al., 2012). Research on thermoregulation and thermal sensitivity has been ongoing for decades, but a major turning point in our understanding of thermoregulation came with the conception of thermal performance curves (or thermal reaction norms) in the 1970s. Thermal performance curves relate some measure of performance to temperature (Huey and Slatkin, 1976; Angilletta, 2009; Huey and Stevenson, 2015) and allow for determination of the temperature at which the performance is maximised (T_opt_) and estimation of the range of temperatures over which the species performs well (performance breath). It is a simple concept that has revolutionised the study of thermoregulation, especially in ectotherms.

The best uses of thermal performance curves are those that explicitly relate variation in function across body temperatures to some performance metric with direct fitness consequences (Angilletta et al., 2006; Schulte et al., 2011; Dowd et al., 2015) that might be virulence in bacteria (Ashrafi et al., 2018), growth and development in insects (Shi et al., 2015), or running performance in lizards (Hertz et al., 1983). At a biochemical and even tissue level, the same basic relationships between body temperature and function should hold for endotherms as well as they do for ectotherms (reviewed in Seebacher and Little, 2017). This realisation led to the proposal that thermal performance curves might also be useful in understanding the variation in body temperature among endotherms and consequences of that variation for coping with environmental conditions (Angilletta et al., 2010). Although the general idea should be transferable from ectotherms to endotherms, the specifics will necessarily be different because body temperature is highly modulated by endotherms through enhanced physiological thermoregulation. In practice, physiological thermoregulation has made measuring thermal performance curves in endotherms exceedingly difficult (Levesque and Marshall, 2021). A few researchers have managed to describe a thermal performance curve for a specific tissue group either *in vitro* (James et al., 2015; Seebacher and Little, 2017) or *in vivo* (Rummel et al., 2018, 2019). Even fewer have successfully measured whole animal performance across temperatures in endotherms (Seymour et al., 1998; Rojas et al., 2012; reviewed in Levesque and Marshall, 2021).

**Figure 1:**
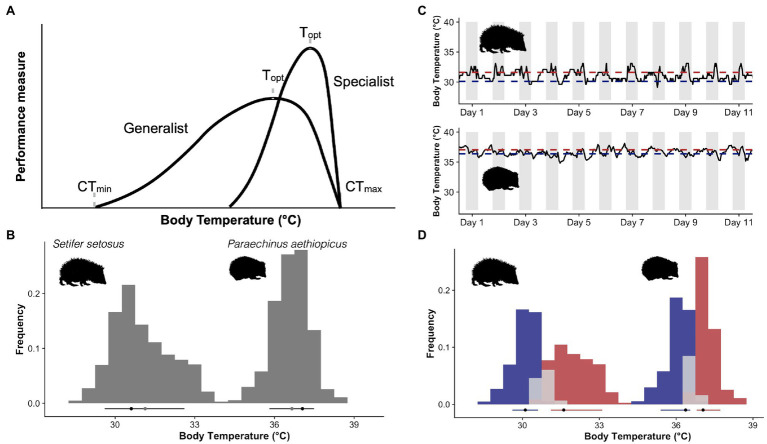
**(A)** Hypothetical thermal performance curves for two ectotherms, a thermal generalist capable of performance over a wide range of temperatures and a specialist with higher performance but a narrower range. **(B)** Core body temperature frequency distribution curves from a greater hedgehog tenrec [*Setifer setosus* (Levesque et al., 2014)] and a desert hedgehog [*Paraechinus aethiopicus* (Boyles et al., 2017)]. Black dots indicate the mode, grey the mean and the lines the limits of the 10th and 90th quantiles of the body temperature distribution. **(C)** Free-ranging body temperature traces for the same two species, grey bars indicate the dark phase as both species are nocturnal, this also indicates the acrophase of their activity cycle. Red lines indicate the mode of the active phase and blue the resting. **(D)** Histograms generated by dividing the body temperature data into active (red) for data above the 55th quantile, resting (blue) for data below the 45th quantile or transitional (grey). Quantiles were generated using additive quantile regression smoothing (see text) to account for potential seasonal changes in the level of body temperature. Black dots indicate the mode, and the lines the 10th-90th quantiles of the distribution. Picture credits: Kerileigh Lobban (tenrec) and Claus Rebler (hedgehog, phylopic.org).

Because thermal performance is difficult to measure directly in endotherms, several authors (including us) have suggested that distributions of body temperature might serve as a proxy for thermal performance (Angilletta et al., 2010; Boyles and Warne, 2013; Levesque and Marshall, 2021). This suggestion is based on the idea that thermoregulation and thermal sensitivity have coadapted in endotherms, as appears to be the case in ectotherms. The coadaptation of thermoregulation and thermal sensitivity should lead to a generalist-specialist trade-off where individuals, populations and species fall along a continuum between strict thermoregulation with high peak performance at the chosen set point temperature and more flexible thermoregulation with moderate performance across a wide range of body temperatures (Angilletta et al., 2003; Figure 1A). Thus, the shape of a distribution of body temperature measurements could theoretically give some indication of the shape of a thermal performance curve (Figure 1B). The obvious benefit of this method of estimating endotherm thermal performance curves in this manner is the (relative) ease with which one can measure body temperature in free-ranging animals as well as the availability of existing datasets. As biologging technologies advance (Chmura et al., 2018; Hawkes et al., 2021), so does our collective ability to describe body temperature distributions. The use of body temperature distributions as a proxy for thermal performance curves could therefore facilitate large-scale examinations of thermal sensitivity across the mammalian and avian phylogenies, which would represent a huge advance in understanding how mammals and birds will respond to climate change.

It is telling that no such analyses exist. In fact, although researchers have found some utility in using thermal performance curves to address theoretical aspects of thermoregulation in endotherms (Boyles et al., 2011; Boyles and Warne, 2013; Levesque and Marshall, 2021), few researchers have used them in empirical research (but see Seebacher and Little, 2017). An inherent limitation of using body temperature distributions as a proxy for thermal performance is that the entire idea is based on an unverified and very difficult to test assumption proposed by Angilletta et al. (2010) that thermoregulation and thermal sensitivity are coadapted in endotherms. This is a significant hurdle, but there are some obvious pathways to advance this research. Physical or pharmacological manipulations of hypothalamic set point temperatures might offer an opportunity to manipulate body temperature and measure relevant functional traits (e.g. enzyme performance, digestive ability or muscle function) under a variety of body temperature conditions. Likewise, regional heterothermy throughout the body means that different tissue groups will be exposed to different thermal conditions and may therefore exhibit different levels of thermal sensitivity (James et al., 2015; Seebacher and Little, 2017). Finally, heterothermic species that have widely variable body temperatures over daily or annual cycles can serve as useful model taxa for studying thermal sensitivity at different body temperatures (Willis and Brigham, 2003; Rojas et al., 2012; Nowack et al., 2016b).

In this perspectives paper, we address the usefulness of body temperature distributions in assessing thermal performance or thermal sensitivity in endotherms. We first provide a brief overview of the primary drivers of variability in body temperature in endotherms and then attempt to find evidence of the potential utility of body temperature distributions to serve as a proxy for thermal performance curves. We do this by comparing the general shape of body temperature distributions from several small mammal species to see if they qualitatively conform to the classical shape of a thermal performance curve.

## What Is Body Temperature?

Body temperature in endotherms is an emergent property of behavioural, morphological and physiological mechanisms that either generate heat or control its loss to the environment (Tattersall et al., 2012; Seebacher, 2020). Physiological heat generation occurs *via* metabolic activity at rest, muscular activity and shivering and non-shivering thermogenesis, among others (Humphries and Careau, 2011; Seebacher, 2018). The maintenance of body temperature within a narrow range of temperatures allows for sustained aerobic activity and independence from environmental conditions and is therefore believed to be the greatest benefit of the evolution of endothermy (Bennett, 1991; Farmer, 2000; Koteja, 2000; Clarke and Pörtner, 2010). Regulated in the hypothalamus, body temperature is defended at a set point by balancing heat production and heat retention with heat dissipation mechanisms (panting, changes in posture, etc.; Romanovsky, 2007; Zhao et al., 2017). Set points can be variable and difficult to assess outside of experiments directly manipulating hypothalamic temperatures (Heller et al., 1977). Furthermore, at any point in time, a particular value for body temperature could be the result of active control, passive cooling, a by-product of heat generated during activity or a transitional value between various states. Regional heterothermy adds an additional axis of variation in body temperature of endotherms and tissue temperature can vary depending on where the measurements are taken (Irving and Krog, 1955; Maloney et al., 2019). For the remainder of the paper, we will be referring predominantly to core body temperature as it is the most commonly measured form of internal body temperature, but we emphasise that core temperatures are not representative of all tissue temperatures.

Although we often speak about the ‘near-constant’ body temperature of endotherms, the range of temperatures shown by any one individual is often wider than realised and can be highly variable across both daily and annual cycles. Most endotherms show a pronounced circadian variation, with a higher body temperature during the active phase (acrophase) and a lower body temperature during the rest phase (scotophase) of their circadian cycle (Aschoff, 1983; Maloney et al., 2019; Refinetti, 2020). The amplitude of circadian rhythms is variable between species and also varies within species or individuals with water and food availability, ambient temperature and reproductive status (Poppitt et al., 1994; Scribner and Wynne-Edwards, 1994; Refinetti, 1999; Hetem et al., 2010; Levesque et al., 2014; Maloney et al., 2017). Differing pressures over evolutionary history have led to myriad thermoregulatory patterns in endotherms of all sizes (Lovegrove, 2012). Thermoregulatory variation is most pronounced in species which reduce body temperature during energy-saving torpor (Grigg et al., 2004; Ruf and Geiser, 2015; Nowack et al., 2020), and the use of daily torpor during the rest phase combined with continued activity during the active phase can lead to pronounced daily amplitudes. Similarly, some endotherms inhabiting warm environments temporarily forgo water-costly cooling mechanisms and allow their body temperature to passively increase with ambient temperature during acute heat to save water (Degen, 2012; Gerson et al., 2019; Reher and Dausmann, 2021).

The vast majority of work on variation in body temperature in endotherms has been either in the context of describing heterothermic and homeothermic patterns or circadian and circannual rhythms. In both of those contexts, body temperature distributions contain useful information, but researchers have largely focused on measures of central tendencies (e.g. the mean of body temperature during normothermy or during the day). Due to the complexities of body temperature regulation in endotherms, assigning a single value (such as a mean) to a species can obscure important information about their underlying physiology. Understanding the distinction between the thermoregulatory physiology of an endotherm during the rest and active phases of their daily cycle (Aschoff, 1981; Wright et al., 2002) is essential to understanding the links between temperature and performance. Selection for high body temperatures during the activity phase (Koteja, 2000; Lovegrove and Mowoe, 2014; Stawski et al., 2017) would not preclude the additional adaptive benefits of energy savings during rest (from normothermic resting to nocturnal hypothermia through to deep hibernation). The distinction between these two very different physiological states (active and resting) as well as the differing determinants of variability in temperature during these states has been lost by focusing predominantly on mean body temperatures. For practical purposes, this thermolability means that mean body temperatures alone are unlikely to contain particularly useful information for understanding the evolution of thermal performance in endotherms. We therefore need methods that help us quantify more than just means.

## Do Body Temperature Distributions Contain Useful Performance Signals?

**Figure 2:**
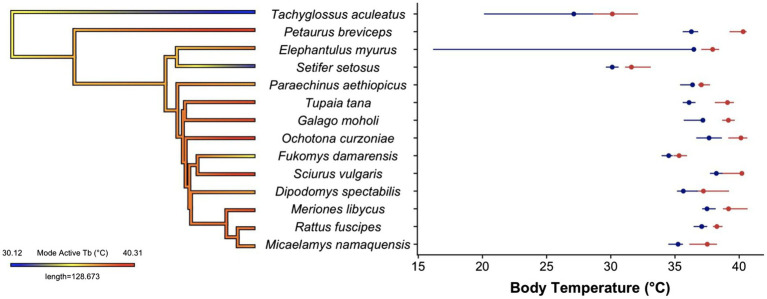
Body temperature distributions for the 14 species of small mammals. The phylogeny shows estimated ancestral states for the mode of the active phase body temperatures. There was no relationship between body temperature and phylogenetic position in this sample. Phylogenetic tree was generated using the *contMap* function in the R package ‘phytools’ (Revell, 2012) using the mammal supertree from Upham et al. (2019). The spread of the active phase (red) and resting phase (blue) are shown for a single individual from each species. The dots indicate the modes of each phase and the daily cycle, and lines represent the 10th–90th quantiles of the distribution. The datasets for *Tachyglossus aculeatus* and *Elephantulus myurus* include torpor use, which leads to a clear left-skewed distribution. Body temperature traces were obtained from the following publications *Dipodomys* (Morales et al., in press), *Elephantulus* and *Micaelamys* (Boyles et al., 2012), *Fukomys* (Streicher et al., 2011), *Galago* (Nowack et al., 2013), *Meriones* (Alagaili et al., 2017), *Ochotona* (Speakman et al., 2021), *Paraechinus* (Boyles et al., 2017), *Petaurus* (Nowack and Geiser, 2016), *Rattus* (Nowack and Turbill unpublished data), *Sciurus* (Dausmann et al., 2013), *Setifer* (Levesque et al., 2014), *Tachyglossus* (Nowack et al., 2016a) and *Tupaia* (Levesque et al., 2018).

The very idea that body temperature distributions might tell us something about thermal sensitivity suggests the shape of the distributions is also important. Beginning with Angilletta et al. (2010), endothermic generalist and specialist thermoregulatory patterns have been depicted as left-skewed, unimodal distributions reminiscent of ectothermic thermal performance curves. This model served as a useful starting point in theoretical discussions of the coadaptation of thermoregulation and thermal sensitivity, but left-skewed, unimodal distributions of body temperatures are far from universal in endothermic species. Instead, bimodal distributions are common and right-skewed distributions do occur in some species (e.g. McKechnie et al., 2007; Levesque et al., 2018; Figure 1B). The disjunction between theoretical treatments of body temperature distributions and reality is obviously problematic. To address whether core body temperature distributions provide a useful analogue to thermal performance curves and to encourage a more biologically relevant means of describing endotherm body temperature distributions, we collated core (intraperitoneal) temperatures for single individuals of 13 different species of small eutherian and marsupial mammals (<300g) and one monotreme (<4,000g). This was not intended to be an exhaustive list but was chosen to represent species from different habitats and from across the mammalian phylogeny. We included only small mammals here to limit the influence of body mass, which should normally be taken into consideration, but is not fundamental to the point we hope to make. We also preferentially included data from active seasons (spring, summer or fall) to focus on times of year when increases in active body temperatures may be linked to increases in performance. We used non-stationary waveform analysis (Levesque et al., 2017) to split active and resting body temperatures in each species. This method has the advantage of being agnostic to both time and the level of body temperature and therefore can account for changes in the level and amplitude of body temperature cycles, such as those seen seasonally or during estrous cycles. We considered all measurements between the 45th and 55th quantiles estimated by the waveform analysis as transitory between active and resting. Measurements that fell above the 55th quantile were classified as active temperatures and those below the 45th quantile were classified as resting temperatures. This avoided problems of defining active periods based on external factors, such as day/night cycles (Figure 1C). For each phase, we then calculated modal temperature as a measure of central tendency, the 10th and 90th quantiles, and a measure of skewness (Bickel, 2002) of active and resting body temperature distributions for each species (Figures 1D, 2).

Despite the limited dataset, our analysis clearly reveals variation in the shape of body temperature distributions between mammalian species (Figure 2). The overall distributions for most (8/14) of the species were right-skewed, the opposite of the normal left skew thermal performance curves observed in ectotherms, tissue assays and protein function (Angilletta, 2009). A right-skewed performance curve indicates mammals have more leeway to allow body temperature to increase above set point than decrease below it. This fits with routine increases in body temperature that have been observed during locomotion in both small mammal (Bieber et al., 2017) and large mammals (Hetem et al., 2019). The shape of the body temperature distribution that combines both resting and active temperatures (e.g. Figure 1B) may, however, be irrelevant because as mentioned before, many mammals have distinctly bimodal body temperature distributions. We originally assumed that if we separate active from resting temperatures, a unimodal, left-skewed distribution may likely fit the active body temperatures of those species with a bimodal distribution. In our sample of small mammals, body temperature distributions display bimodality for most (8/14) of the species included (indicated by a Hartigan’s dip test statistic <0.05). However, the resulting activity and resting distributions, while at least no longer bimodal, still did not follow the shape of a classic ectotherm thermal performance curve (Figure 2). Instead, most (6/8) of the species with a bimodal body temperature distribution also had a right-skewed body temperature distribution of active body temperatures. Clear left-skewed distributions were, however, observed for species with data spanning periods of torpor use (e.g. *Elephantulus, Galago and Tachyglossus*), which provides an interesting avenue for future research.

Our cursory inter-species comparison also revealed surprisingly comparable between-species variability in modal active and resting body temperatures (activity mode 37.2°C, mean 37.4°C, st. dev. 3.1°C, range 30.1–40.3°C; resting: mode 35.6°C, mean 35.4°C, st. dev. 3.1°C, range 27.1–38.2°C), although active temperatures were consistently higher than resting (Figure 2). This may, however, be influenced by our choice of species, and the time of year at which the recordings were taken, although our values do span the range of reported values for mammalian body temperatures (Clarke and Rothery, 2008). When given the option, we preferentially chose values from the active season to avoid additional complications of differences in torpor use, but in some species (*Elephantulus, Setifer and Tachyglossus*), torpor is unavoidable. Had we included data for the full annual cycle, we would expect to find additional differences in the level and potentially the shape of the distributions depending on factors, such as reproduction, time of year and environmental conditions. The intra-species difference for the daily amplitude between active and resting modes of our 14 species showed a wide variation (mode: 1.6°C, mean: 2.0°C, st. dev. 0.9°C, range 0.68–4.03°C), with the hedgehog (*Paraechinus aethiopicus*) showing the least amount of difference between active and resting and the sugar glider (*Petaurus breviceps*) displaying the largest daily amplitude, yet these might be expected to change had we focused on different times of year. These findings demonstrate the utility of body temperature distributions in generating points of comparison for eco-physiological or comparative studies.

## Conclusion

Although our limited sample of species is small, mostly stems from small-bodied mammals and is biased towards our own study systems, we found no evidence to suggest that using distributions of core body temperatures is likely to be fruitful as proxies for ectotherm thermal performance curves. This does not mean that endotherms are free from the biophysical constraints that dictate temperature effects on performance, and therefore the classical shape of ectotherm thermal performance curves. Instead, it only suggests that core body temperature distributions are not a good proxy for the shape of thermal performance curves in endotherms. In fact, they might still be useful for estimating the single temperature at which most performances are maximised (the optimal temperature, possibly represented here by the mode of the active phase), just not for describing the thermal sensitivity of a species. We also still expect to find the left-skewed thermal performance curves, so common in ectotherms, in endotherms at cellular, tissue and whole organism levels (Seebacher and Little, 2017). We only conclude here that those performance curves will likely need to be measured directly at those levels instead of estimated indirectly *via* core body temperature distributions.

We also do not wish to discourage the description and use of body temperature distributions in the study of endotherm thermoregulation. Fully describing body temperature distributions will undoubtedly lead to advances that would not be possible by focusing only on measures of central tendencies. Body temperature distributions may be more valuable in addressing ecological questions about how a mammal or bird interacts with its environment than as direct analogues to ectotherm thermal performance curves. In fact, our cursory analysis hints that there is some benefit in distinguishing between the distinct active and resting phases of the daily body temperature cycle, at least at an interspecific comparative level, which should prove fruitful in evolutionary studies. There is also potential in the ability to analyse both the level and variability of active and resting temperatures separately which should prove beneficial in both within- and between-species studies. We strongly encourage the use of more detailed body temperature data so that we can move beyond the idea of endotherms as strict homeotherms and embrace the true heterothermic diversity of extant endotherms.

## Data Availability Statement

The raw data supporting the conclusions of this article will be made available by the authors, without undue reservation.

## Author Contributions

DLL and JN conceived the idea and outlined the manuscript. DLL and JB designed the analysis and prepared the figures for the manuscript. All authors contributed to the article and approved the submitted version.

## Funding

DLL was supported by the USDA National Institute of Food and Agriculture, Hatch project number 21623 through the Maine Agricultural & Forest Experiment Station.

## Conflict of Interest

The authors declare that the research was conducted in the absence of any commercial or financial relationships that could be construed as a potential conflict of interest.

## Publisher’s Note

All claims expressed in this article are solely those of the authors and do not necessarily represent those of their affiliated organizations, or those of the publisher, the editors and the reviewers. Any product that may be evaluated in this article, or claim that may be made by its manufacturer, is not guaranteed or endorsed by the publisher.
